# Translation and cross-cultural adaptation of the *Identification des patients nécessitant des soins PALLiatifs généraux et spécialisés* (ID-PALL©) to Brazilian Portuguese

**DOI:** 10.1017/S1478951526103265

**Published:** 2026-08-04

**Authors:** Fernanda Ilha Pedroso, Etiane de Oliveira Freitas, Luis Felipe Dias Lopes, Patrícia Bitencourt Toscani Greco, Graciela Dutra Sehnem, Sahar Obeid, Carlos Laranjeira, Silvana Bastos Cogo

**Affiliations:** 1 Universidade Federal de Santa Maria, Santa Maria, RS, Brazil; 2 Universidade Federal do Rio Grande, Rio Grande, RS, Brazil; 3 Department of Psychology and Education, School of Arts and Sciences, Lebanese American University, Jbeil, Lebanon; 4 School of Health Sciences, Polytechnic University of Leiria, Leiria, Portugal; 5 Centre for Innovative Care and Health Technology (ciTechCare), Polytechnic University of Leiria, Leiria, Portugal; 6 Comprehensive Health Research Centre (CHRC), University of Évora,Évora, Portugal

**Keywords:** palliative care, patient care management, translations, validation studies, nurses

## Abstract

**Objectives:**

The growing demand for palliative care (PC) services and the need for standardized approaches to patient identification highlight the importance of having validated tools available in Brazilian Portuguese. The absence of culturally adapted instruments may hinder appropriate patient assessment, delay referral to PC services, and ultimately compromise the quality of care delivered. This study aims to translate and adapt the *Identification des patients nécessitant des soins PALLiatifs généraux et spécialisés* (ID-PALL©) instrument into Brazilian Portuguese.

**Methods:**

Herdman’s recommendations were followed, which include conceptual, item, and semantic validation stages. After authorization from the ID-PALL© developers, the translation, synthesis of the translated versions, and back-translation of the instrument were performed. Subsequently, a committee of 11 experts evaluated the semantic, idiomatic, conceptual, and cultural equivalence between the versions, resulting in the development of the pre-final version. To validate the instrument’s content, this version was pre-tested (*n* = 30) with the participation of physicians and nurses. For statistical analyses, the content validity coefficient (CVC) was calculated.

**Results:**

The cross-cultural adaptation demonstrated the suitability of the translated versions after semantic and cultural adjustments. The pre-final version showed satisfactory comprehension and semantic (CVC), idiomatic (CVC), conceptual (CVC), and cultural (CVC) equivalence, which enabled the development of the final version, named IDPALL-BR. The data obtained in the pre-test demonstrated content validity among the target audience, with a CVC = 0.909.

**Significance of results:**

The Brazilian Portuguese version of this instrument has semantic validity and, therefore, shows potential for screening general and specialized PC needs.

## Introduction

Loss of functionality and quality of life are common to many acute and chronic diagnoses and are intrinsically related to challenging prognoses, dependence on care, and threats to life (Kelley and Bollens-Lund 2018). Palliative care (PC) aims to improve and relieve suffering and improve the quality of life of people with life-threatening illnesses and their families by the early identification, correct assessment, and treatment of pain and other biopsychospiritual and social signs and symptoms (WHO 2020). When established in a timely and appropriate manner, this care model promotes the rational use of healthcare resources by reducing interventions that are clinically unnecessary for the patient being cared for (Hui et al. 2022; D’Alessandro 2023).

Although it is a specialized approach, PC should be offered at all levels of healthcare and, therefore, provided by non-specialist teams who have been properly trained (Baylis et al. 2023). General PC care constitutes the foundation of this care model (D’Alessandro 2023), since the demand for this type of care far exceeds the capacity of specialized teams (Santos et al. 2020; Vitorino et al. 2023).

Globally, there are countries and regions (such as North America, Europe, and Australia) where PC is governed by specific legislation, but more than 70% of PC needs occur in developing countries (WHPCA 2020). According to the map developed by the World Hospice and Palliative Care Alliance, Brazil is classified as 3b on a 6-point scale, which indicates limited availability of morphine, limited number of PC services, and a lack of awareness among the general public and professionals regarding PC (Clark et al. 2020).

In 2024, the modern PC movement in Brazil reached a significant milestone with the establishment of the National Palliative Care Policy, aimed at addressing gaps in PC provision through the creation of PC teams and PC education for healthcare professionals and the general population. Under this policy, simpler cases should be managed by these teams, while complex cases should receive specialized care (Brasil 2024).

From this perspective, non-specialist professionals and teams are responsible for general care needs, including the initial management of pain and other symptoms; the basic approach to cases of depression and anxiety; and conducting essential discussions related to prognosis, therapeutic options, suffering, and welcoming family members (IAHPC 2018; Radbruch et al. 2020; Hui et al. 2022).

In turn, specialists are responsible for tasks related to the care of more complex cases, including the management of difficult-to-control symptoms; treating situations of complex depression, complicated grief, and existential distress; mediating conflicts between family members and teams regarding therapeutic goals or methods; analyzing potential scenarios of futile treatment; managing indicators; and providing continuing education and training for other professionals at all levels of healthcare (IAHPC 2018; Radbruch et al. 2020; Hui et al. 2022; ANCP 2023).

This study emerged from the need to screen and stratify the care needs of people with life-threatening illnesses, according to their complexity, aiming to facilitate the timely implementation of person-centered interventions, preferably from the early stages of the disease, and not restricted to end-of-life contexts. Although there are already validated needs-assessment tools for the Brazilian context, such as the *Supportive & Palliative Care Indicators Tool* (SPCIT-BR™) (Edinburgh 2019) and NECPAL-BR (Santana et al. 2020), these do not assess the demand for general and specialized PC. The adaptation, at a national level, of a tool that can be applied by nurses and physicians, based on their clinical observations, constitutes a useful tool for stratifying PC needs.

Therefore, the objective of this research was to carry out the translation and cross-cultural adaptation (CCA) of the *Identification des patients nécessitant des soins PALLiatifs généraux et spécialisés* (ID-PALL©) for the Brazilian context. This tool will enable healthcare professionals to more accurately identify patients with PC needs, promote earlier integration of palliative approaches, and contribute to improving person-centered care outcomes. Furthermore, this initiative supports the broader development of PC practices in Brazil by providing a scientifically validated tool aligned with international standards.

## Method

### Study design

This is a descriptive and methodological study focused on the CCA of the original French version of the ID-PALL© instrument (Teike Lüthi et al. 2020) into Brazilian Portuguese, as well as the assessment of its semantic equivalence in this language. The study was conducted in a Hospital Unit located in the central region of Rio Grande do Sul, Brazil, between January 2025 and January 2026. Ethical approval was obtained from the Research Ethics Committee of the Federal University of Santa Maria (UFSM) (CAAE: 85729325.3.0000.5346; approval number: 7344518).

### Procedures

**Figure 1.**
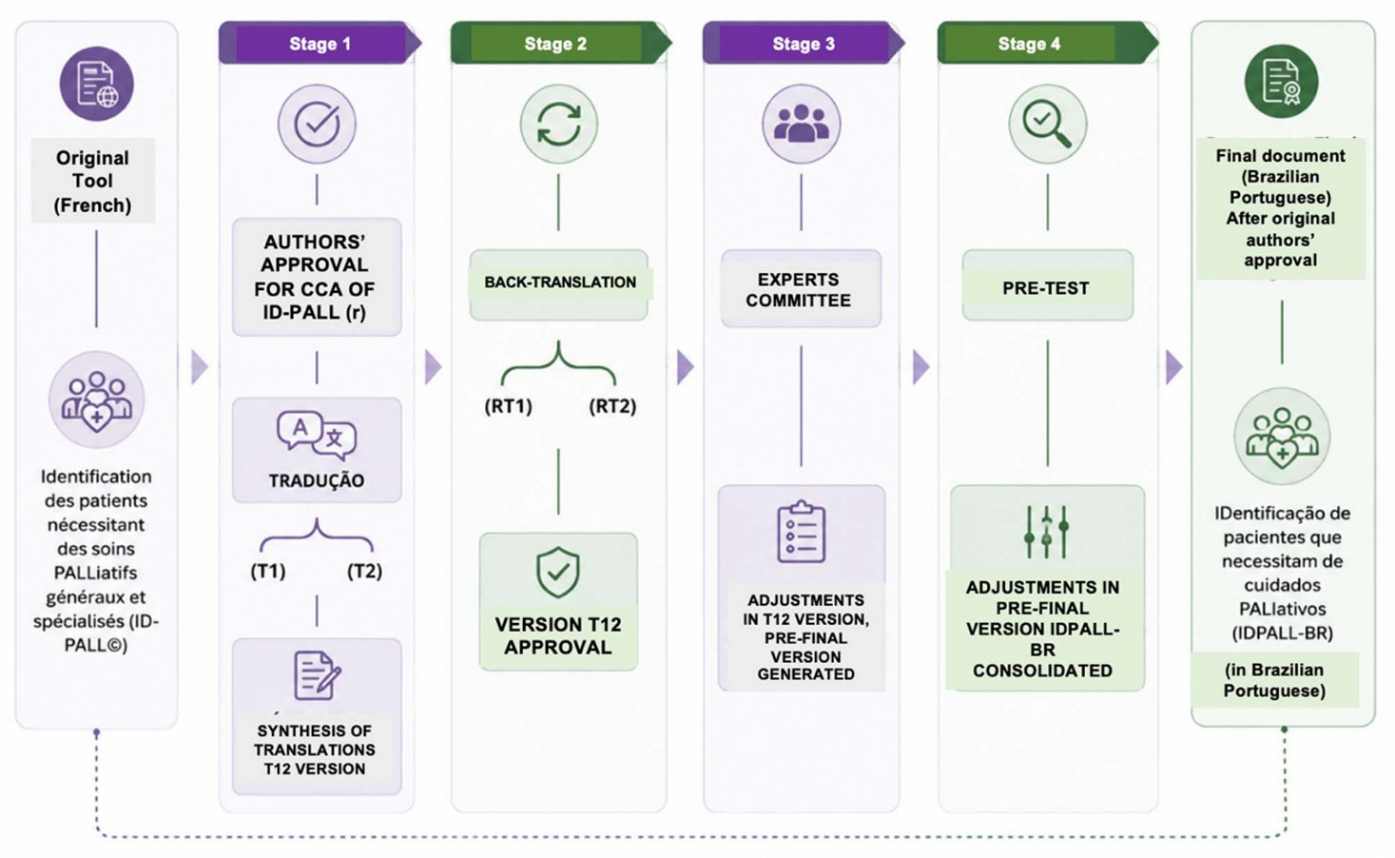
Flowchart of the translation and cross-cultural adaptation process of the ID-PALL©. Figure 1 long description.

The study was conducted based on the Herdman recommendations (Herdman et al. 1997), as shown in Figure 1.

### Study stages

#### Stage 1 – Translation and synthesis of the translations into Brazilian Portuguese

The translation was performed by 2 bilingual translators (T1 and T2), both of whom are native speakers of Brazilian Portuguese, Brazilian nationals, and residents of Brazil; one holds a degree in humanities, and the other holds a Ph.D. in nursing. They translated the instrument from French into Portuguese independently. They also translated the general PC practice recommendations accompanying the instrument. Subsequently, a synthesized version (T12) was developed by the study team and translators.

#### Stage 2 – Back-translation

Two bilingual translators (RT1 and RT2), whose native language is French, back-translated the T12 version from Portuguese into French, without knowledge of the original instrument. The 2 back-translated versions were then compared with the original version. In the absence of relevant discrepancies, version T12 was forwarded to the instrument’s developers, who approved the study’s continuation without revisions.

#### Stage 3 – Expert committee

To conduct the expert evaluation, we invited 20 experts representing all 5 regions of the country (Coluci et al. 2015; Marconi and Lakatos 2018). Only 11 participated, covering 4 regions of Brazil, all of whom are specialists in 2 areas critical to this study: PC and methodological studies. After signing the informed consent form (ICF), the experts received the original version of the ID-PALL©, as well as the versions generated by the translators (T1, T2, RT1, RT2) and version T12, which served as the basis for this stage.

The evaluation form had some initial questions regarding the sociodemographic profile of the experts. They were then asked to evaluate each item of the translated instrument for semantic, idiomatic, conceptual, and cultural equivalence with the original version, using a Likert score from 1 to 4, indicating no equivalence, little equivalence, good equivalence, and fully equivalent, respectively. Experts were asked to provide suggestions for improving items with a score of 1 or 2. After the necessary adjustments, the pre-final version was established. There was no need for a second round because this version presented a satisfactory content validity coefficient (CVC) (Hernández-Nieto 2002).

#### Stage 4 – Pre-test

The pre-test was conducted in 3 adult inpatient units of a large university hospital in the central region of Rio Grande do Sul. Patient recruitment was carried out by the principal investigator in collaboration with the unit’s staff. The inclusion criteria was having any medical diagnosis of a life-threatening disease, regardless of underlying etiology, sex, or age group. For convenience, 5 patients with chronic progressive diseases were selected; they all agreed to participate in the study and signed the patient ICF. Using the pre-final version of the ID-PALL, each patient was assessed by 6 healthcare professionals (physicians and nurses) who agreed to participate in the study and signed the professional ICF. They all had at least 6 months of experience in caring for chronic patients.

The sample totaled 30 professionals, in accordance with the recommendations for the pre-test (Beaton et al. 2007). The target population was healthcare professionals, since the evaluation focused on their understanding and acceptance of the instrument to identify the demand for PC and direct patients to general or specialized care.

The pre-final version of the ID-PALL was administered independently, based on the evaluating professional’s clinical assessment of the patient’s medical records pertaining to the first 48 hours of the current hospitalization (Teike Lüthi et al. 2020). The instrument was evaluated by assigning a Likert scale score from 1 to 4, corresponding to no understanding, scarcely understandable, fairly understandable, and fully understandable, respectively. Additionally, the evaluation form contained 4 questions to determine the sociodemographic characteristics of the participants.

After analyzing the pre-test results, minor adjustments were made to the pre-final version based on suggestions by participants, resulting in the instrument’s final version, named IDPALL-BR. A second round was not necessary due to a satisfactory CVC (Hernández-Nieto 2002). The cross-culturally adapted version was sent to the study developers and received the authors’ approval without reservation.

### Data analysis

In steps 4 and 5, the same procedure was followed: data were entered into Excel spreadsheets, with independent double-entry. After correcting typing errors and inconsistencies, statistical analysis was performed; for semantic equivalence, the CVC (Hernández-Nieto 2002) was calculated for each item assessed; a *score* ≥0.80 was considered an acceptable agreement. All tests were performed using the software R, IBM (SPSS), version 21, and Excel 2010 (Microsoft Office). Descriptive analysis was used for data characterizing the study participants, with categorical and quantitative variables expressed in absolute and relative frequencies.

## Results

Authorization for the translation and CCA of the ID-PALL© was obtained via email contact with the instrument’s developers, who consented and made the instrument available in its original form. The ID-PALL scale was independently translated by 2 bilingual translators. Following the independent translations, a translation consensus meeting was held to discuss aspects aimed at ensuring the best semantic fit rather than a literal translation of the terms.

At that point, both translators discussed the term “les proches,” which translates to “relatives,” and raised the point that many patients are not cared for by blood relatives, suggesting the use of the term “support network.” However, based on the concept of PC, the study opted to use the term “family members.” Furthermore, items 2.1 and 3 (ID-PALL G) and items 3, 4, 6, and 7 (ID-PALL E) presented some difficulty and/or uncertainty for the translators.

In item 2.1, the term “*escarre*” is literally translated to Portuguese as “escara,” which is synonymous with “pressure ulcer”; however, both terms are no longer recommended in clinical practice. Thus, the term “pressure injury” was adopted since this is the appropriate terminology to more accurately describe “any lesion on intact or ulcerated skin” (SOBEST/SOBENDE 2016).

In item 3, the term “*ventilation artificielle*” is translated literally as “artificial ventilation.” Although understandable and correct, it was decided to replace it with “mechanical ventilation,” a terminology that is commonly used in Brazil. Furthermore, in item 3 of the ID-PALL E domain, the term “*le code*” is translated, in Brazilian Portuguese, as “código,” which means “collection/system of laws, rules, precepts, and formulas,” but this was changed to the Portuguese word “protocolo” (protocol), which better reflects a standardized guideline that directs the decisions and conduct of healthcare professionals.

In item 6, the term “*envisagée*” is literally translated as “considerado,” which means “taken into account, judged, regarded”; however, given the context of palliative sedation, the term was changed to “planejada” (planned).

The summary version (T12) was back-translated by 2 native French-speaking translators residing in Brazil, who are fluent in Portuguese and have a background in healthcare but no connection to PC. The back-translations required no adjustments, and the T12 version was validated.

Subsequently, version T12 was approved by the authors and submitted to the subsequent evaluation stage (Table 1).

**Table 1.** Original version and T12 version Table 1 long description.

Versão Original ***ID-PALL G***	Versão T12 ***ID-PALL G***
*1. Seriez-vous surpris si ce patient décédait dans les 12 prochains mois ?*	1. Você ficaria surpreso se este paciente morresse nos próximos 12 meses?
*2. Patient atteint d’une maladie évolutive ou d’un ensemble de maladies/co-morbidités qui **limite son espérance de vie*** ***ET*** *qui présente (plusieurs choix possible):* ***un déclin fonctionnel général*** *(avec réversibilité limitée et augmentation du besoin de soutien pour les activités de la vie quotidienne)*	2. O paciente possui uma doença progressiva ou um conjunto de doenças/comorbidades que **limitam sua expectativa de vida** **E** apresenta (é possível escolher mais de uma resposta): **Um declínio funcional geral** (com reversibilidade limitada e aumento da necessidade de suporte para as atividades de vida diárias)
*2.1 **une instabilité marquée durant les 6 derniers mois** (définie par: un symptôme non contrôlé du point de vue du patient OU une escarre catégorie ≥3 OU plus qu’un épisode d’état confusionnel aigu, d’infection, d’hospitalisation non programmée ou de chute)*	2.1 **uma instabilidade acentuada durante os últimos 6 meses** (definida por: um sintoma não controlado do ponto de vista do paciente OU uma lesão por pressão categoria ≥3 OU mais de um episódio de confusão mental aguda, de infecção, de hospitalização não programada ou de queda)
*2.2 **une souffrance psychosocial e ou existentielle** (du patient ou des proches)*	2.2 **Sofrimento psicossocial ou existencial** (do paciente ou da sua família)
*2.3 le besoin **d’un accompagnement pour la prise de décisions** lors de la dernière phase de la vie*	2.3 a necessidade **de acompanhamento para tomar decisões** acerca da fase final de vida
*3. Interruption, effective ou envisagée, des traitements à visée curative ou des mesures de **soutien vital** (ex: ventilation artificielle, dialyse, alimentation et/ou hydratation artificielle)*	3. Interrupção, efetiva ou planejada, dos tratamentos curativos ou das medidas de **suporte vital** (ex: ventilação mecânica, diálise, alimentação e/ou hidratação)
*4. Demande de **soins de confort/palliatifs** par le patient, les proches ou les professionnels*	4. Demanda de cuidados de conforto/paliativos pelo paciente, família e/ou profissionais envolvidos.
Versão Original ***ID-PALL E***	Versão T12 ***ID-PALL E***
*1. Présence **d’au moins un symptôme sévère et persistent**, y compris la douleur, n’ayant pas répondu de manière satisfaisante au traitement dans un délai de 48 h*	1. Presença **de ao menos um sintoma severo e persistente**, incluindo a dor, não respondendo de maneira satisfatória ao tratamento em um período de 48 h
*2. **Difficultés à évaluer** les symptômes physiques ou les problématiques psychologiques, sociales ou spirituelles*	2. **Dificuldades de avaliar** os sintomas físicos ou os problemas psicológicos, sociais e espirituais
*3. **Désaccord ou incertitude** chez le patient, les proches ou les professionnels concernant p. ex. les traitements médicaux, le code de réanimation ou des décisions complexes*	3. **Desacordo ou incerteza** do paciente, família ou dos profissionais em relação, por exemplo, aos tratamentos médicos, ao protocolo de reanimação ou às decisões complexas
*4. **Souffrance psychosociale ou existentielle sévère** chez le **patient*** *(ex: symptômes anxieux ou dépressifs importants, sentiment d’isolement/d’être un fardeau, perte de sens/d’espoir, désir de mort, demande de suicide assisté)*	4. **Sofrimento psicossocial ou existencial severo** do **paciente** (ex: sintomas importantes de ansiedade ou depressão, sentimento de isolamento/de ser um fardo, perda do sentido da vida/de esperança, desejo de morrer, pedido de suicídio assistido)
*5. **Souffrance psychosociale ou existentielle sévère** chez les **proches** (ex: symptômes anxieux ou dépressifs importants, sentiment d’épuisement majeur, déstabilisation importante du système familial, perte de sens/d’espoir)*	5. **Sofrimento psicossocial ou existencial severo** de **familiares** (ex: sintomas importantes de ansiedade ou depressão, sentimento de grande exaustão, desestabilização significativa do sistema familiar, perda do sentido da vida/de esperança)
*6. **Sédation palliative** envisagée (= soulagement d’un symptôme réfractaire et intolérable par une diminution de l’état de conscience à l’aide d’une médication spécifique)*	6. **Sedação paliativa** planejada (= alívio de um sintoma refratário e intolerável por uma diminuição do estado de consciência com a ajuda de uma medicação específica)
*7. **Projet de soin anticipé** ou **directives anticipées** difficiles à établir avec le patient et/ou les proches*	7. **Plano de cuidado antecipado** ou **diretivas antecipadas** difíceis de serem estabelecidas com o paciente e/ou os familiares
*8. Le patient, ses proches ou les professionnels pourraient, selon vous, bénéficier de l’**intervention de spécialistes en soins palliatifs***	8. O paciente, seus familiares ou os profissionais envolvidos podem, na sua opinião, se beneficiarem da intervenção **de especialistas em cuidados paliativos**

In total, 11 experts participated, 55.55% of whom were female. In terms of profession, physicians constituted the largest group (63.64%), while the remaining participants were nurses (36.36%). Regarding the length of experience in their respective fields, 7 (63.64%) participants had more than 5 years of experience in PC or the method. Regarding the highest level of education, 5 (45.45%) held a doctorate, 5 (45.45%) held a master’s degree, and only 1 held a specialization degree (9.10%). Additionally, 3 professionals (27.27%) worked in both clinical practice and teaching concurrently.

Each expert had access to the entire process of this CCA (including versions T1, T2, T12, RT1, and RT2). Following the individual evaluation of each expert’s input, an *Excel* document was generated; in this document, the evaluations and suggestions regarding aspects of semantic, idiomatic, cultural, and conceptual equivalence were compiled to enable statistical analysis.

Through statistical analysis of the data collected in this stage, a CVCt of 0.95 was obtained (Table 2). Although all items had a CVCc ≥ 0.80, adjustments were made in accordance with the experts’ suggestions, as these were perceived by the research team as significant for ensuring greater equivalence; thus, the pre-final version of this CCA was generated (Table 3).

**Table 2.** CVC experts (*n* = 11) Table 2 long description.

Item	Semantic			Idiomatic			Cultural			Conceptual			
	AVG	ERROR	CVCc	AVG	ERROR	CVCc	AVG	ERROR	CVCc	AVG	ERROR	CVCc	CVCt
1	4.00	0.00	1.00	4.00	0.00	1.00	4.00	0.00	1.00	4.00	0.00	1.00	1.00
2	4.00	0.00	1.00	4.00	0.00	1.00	4.00	0.00	1.00	4.00	0.00	1.00	1.00
3	3.98	0.00	0.95	9.82	0.00	0.95	3.82	0.00	0.95	3.73	0.00	0.93	0.95
4	3.91	0.00	0.98	3.82	0.00	0.95	3.82	0.00	0.95	3.82	0.00	0.95	0.96
5	3.91	0.00	0.98	3.82	0.00	0.95	4.00	0.00	1.00	3.82	0.00	0.95	0.97
6	3.91	0.00	0.98	3.73	0.00	0.93	3.73	0.00	0.93	3.64	0.00	0.91	0.94
7	3.82	0.00	0.95	3.73	0.00	0.93	3.82	0.00	0.95	3.73	0.00	0.93	0.94
8	3.91	0.00	0.98	3.73	0.00	0.93	3.91	0.00	0.98	3.82	0.00	0.95	0.96
9	3.91	0.00	0.98	3.91	0.00	0.98	3.82	0.00	0.95	3.91	0.00	0.98	0.97
10	3.91	0.00	0.98	3.91	0.00	0.98	4.00	0.00	1.00	3.82	0.00	0.95	0.98
11	3.82	0.00	0.95	3.64	0.00	0.91	3.45	0.00	0.86	3.55	0.00	0.89	0.90
12	3.73	0.00	0.93	3.55	0.00	0.89	3.73	0.00	0.93	3.55	0.00	0.89	0.91
13	3.91	0.00	0.98	3.91	0.00	0.98	3.82	0.00	0.95	3.64	0.00	0.91	0.95
14	3.82	0.00	0.95	3.82	0.00	0.95	3.64	0.00	0.91	3.64	0.00	0.91	0.93
15	3.82	0.00	0.95	3.91	0.00	0.98	4.00	0.00	1.00	3.91	0.00	0.98	0.98
CVCT			0.97			0.95			0.95			0.94	

CVC: content validity coefficient.

**Table 3.** Version T12 and pre-final version only for questions that underwent changes in this process Table 3 long description.

Version T12	Pré-final version
**Sofrimento psicossocial ou existencial severo do paciente** (ex: sintomas importantes de ansiedade ou depressão, sentimento de isolamento/de ser um fardo, perda do sentido da vida/de esperança, desejo de morrer, pedido de suicídio assistido)	**Sofrimento psicossocial ou existencial grave no paciente** (ex: sintomas intensos de ansiedade ou depressão, sentimento de isolamento/de ser um fardo, perda do sentido da vida/de esperança, desejo de morrer)
**Sofrimento psicossocial ou existencial severo de familiares** (ex: sintomas importantes de ansiedade ou depressão, sentimento de grande exaustão, desestabilização significativa do sistema familiar, perda do sentido da vida/de esperança)	**Sofrimento psicossocial ou existencial grave nos familiares** (ex.: sintomas intensos de ansiedade ou depressão, sentimento de grande exaustão, desestabilização significativa do sistema familiar, perda de sentido/esperança)
**Plano de cuidado antecipado ou diretivas antecipadas** difíceis de serem estabelecidas com o paciente e/ou os familiares	**Planejamento antecipado de cuidados (PAC) ou Diretivas antecipadas de vontade (DAV)** difíceis de serem estabelecidas com o paciente e/ou os familiares

The term “important,” used in the instrument, was replaced with “intense” to avoid prescribing what is or is not important to the patient and their family, as well as to avoid associating this term with anything potentially pejorative. Furthermore, the terms “Advance Care Plan” and “Advance Directives” were replaced by “Advance Care Planning (ACP)” and “Advance Directives of Will (ADW)” to better align with the terminology used in Brazilian clinical practice.

The term “assisted suicide” was removed from the instrument since, under Brazilian law, this practice is not permitted (Brasil 2025). This term is used by the ID-PALL instrument due to its Swiss origin, a country where this practice is regulated. Since it is not applicable in Brazil, the term was removed without prejudice to the Brazilian version.

Thirty professionals participated in the pre-test, including 20 nurses and 10 physicians. Of these, 19 (63.33%) were female, and 11 (36.67%) were male. Regarding years of experience, 21 (70.01%) participants had more than 5 years of experience, with 3 (10%) having more than 20 years of experience. Regarding the highest level of education, 15 (50%) participants had completed a specialization program, 10 (33.33%) held a master’s degree, and 2 (6.67%) held a doctoral degree.

After statistical analysis of the data collected in this stage, a CVCt of 0.909 was obtained (Table 4). Although all items had an acceptable CVCc, 2 questions in the instrument were adjusted based on suggestions from the professionals (target audience) in order to ensure greater equivalence.

**Table 4.** CVC pre-test (*n* = 30) Table 4 long description.

	CVC			
ITEM	Avg	CVCi	Error	CVCc
1	3.80	0.95	4.85694E − 45	0.95
2	3.567	0.892	4.857E − 45	0.892
3	3.433	0.858	4.8569E − 45	0.858
4	3.667	0.917	4.857E − 45	0.917
5	3.667	0.917	4.857E − 45	0.917
6	3.267	0.817	4.857E − 45	0.817
7	3.50	0.875	4.8569E − 45	0.875
8	3.70	0.925	4.8569E − 45	0.925
9	3.667	0.917	4.8569E − 45	0.917
10	3.70	0.925	4.8569E − 45	0.925
11	3.733	0.933	4.8569E − 45	0.933
12	3.733	0.933	4.8569E − 45	0.933
13	3.70	0.925	4.8569E − 45	0.925
14	3.60	0.90	4.8569E − 45	0.90
15	3.833	0.958	4.857E − 45	0.958
CVCt	3.638	0.909	0.000	0.909

CVC: content validity coefficient.

At this point, the adjective “clinical” was added to item 2.2 to clarify the type of instability to which it refers. In item 3, the term “curative treatment” was replaced by “disease-modifying treatment,” which is more appropriate to the context of clinical practice as well as PC.

After making the necessary adjustments, not only to the items but also to the presentation of the instrument, the final version was produced, named IDPALL-BR (Table 5).

**Table 5.** IDPALL-BR Table 5 long description.

ID-PALL G	
IDentificação de pacientes que necessitam de cuidados PALiativos Gerais	
1. Você ficaria surpreso se este paciente morresse nos próximos 12 meses ?	□ Sim □ Não
2. O paciente possui uma doença progressiva ou um conjunto de doenças/comorbidades que **limitam sua expectativa de vida**	□ Sim □ Não
**E** apresenta (é possível escolher mais de uma resposta) :	
2.1 **Um declínio funcional geral** (com reversibilidade limitada e aumento da necessidade de suporte para as atividades de vida diárias)	
OU	□ Sim □ Não
2.2 **uma instabilidade acentuada clínica durante os últimos 6 meses** (definida por: um sintoma não controlado do ponto de vista do paciente OU uma lesão por pressão categoria ≥3 OU mais de um episódio de confusão mental aguda, de infecção, de hospitalização não programada ou de queda)	
OU	□ Sim □ Não
2.3 **Sofrimento psicosocial ou existencial** (do paciente ou da sua familia)	
OU	
2.4 Apresenta necessidade **de acompanhamento para tomar decisões** acerca da fase final de vida	□ Sim □ Não
3. Interrupção, efetiva ou planejada, dos tratamentos modificadores do curso da doença ou das medidas de **suporte vital** (ex: ventilação mecânica, diálise, alimentação e/ou hidratação)	□ Sim □ Não
4. Demanda de cuidados de conforto/paliativos pelo paciente, família e/ou profissionais envolvidos	□ Sim □ Não
ID-PALL E	
IDentificação de pacientes que necessitam de cuidados PALiativos Especializados	
1. Presença **de ao menos um sintoma severo e persistente**, incluindo a dor, não respondendo de maneira satisfatória ao tratamento em um período de 48h	□ Sim □ Não
2. **Dificuldades de avaliar** os sintomas físicos ou os problemas psicológicos, sociais e espirituais	□ Sim □ Não
3. **Desacordo ou incerteza** do paciente, família ou dos profissionais em relação, por exemplo, aos tratamentos médicos, ao procoloco de reanimação ou às decisões complexas	□ Sim □ Não
4. **Sofrimento psicossocial ou existencial grave no paciente** (ex: sintomas intensos de ansiedade ou depressão, sentimento de isolamento/de ser um fardo, perda do sentido da vida/de esperança, desejo de morrer).	□ Sim □ Não
5. **Sofrimento psicossocial ou existencial grave nos familiares** (ex.: sintomas intensos de ansiedade ou depressão, sentimento de grande exaustão, desestabilização significativa do sistema familiar, perda de sentido/esperança)	□ Sim □ Não
6. **Sedação paliativa** planejada (= alívio de um sintoma refratário e intolerável por meio da redução do nível de consciência com o uso de medicação específica)	□ Sim □ Não
7. **Planejamento Antecipado de Cuidados (PAC)** ou **Diretivas Antecipadas de Vontade (DAV)** difíceis de serem estabelecidas com o paciente e/ou os familiares	□ Sim □ Não
8. O paciente, seus familiares ou os profissionais envolvidos podem, na sua opinião, se beneficiar da intervenção **de especialistas em cuidados paliativos**	□ Sim □ Não

## Discussion

People with chronic life-threatening illnesses face many challenges, and these problems frequently encompass the biopsychosocial, spiritual, and financial contexts, highlighting the significant need for PC and its early introduction (Alnajar et al. 2025).

This study developed the translation and CCA of a screening instrument for general and specialized PC needs in the Brazilian context. During the semantic equivalence phase, 3 versions of the instrument were generated, leading to the final version. The results of this study demonstrate that the IDPALL-BR is reliable and valid for screening and stratifying patients regarding their need for general or specialized PC, confirming semantic, idiomatic, cultural, and conceptual equivalence. Furthermore, one of the main findings of this study was the confirmation of semantic equivalence, as evidenced by a CVC of 0.909.

Although none of the calculated CVCs were unacceptable, some changes were justified, since this is a specialized and still underutilized field. Suggestions relevant to clinical practice were adopted, seeking to mitigate difficulties in using the instrument. Despite the legal discrepancies between Brazil (Brasil 2025) and Switzerland (Hurst and Mauron 2003) regarding PC, only one aspect needed to be removed (patient’s request for assisted suicide), causing no harm to the item or the instrument.

This study used the in-hospital setting as its field of application, as the instrument was developed in this environment; however, considering the recommendations of the developers themselves (Teike Lüthi et al. 2020), it should also be validated in other healthcare settings, including the home.

The IDPALL-BR offers benefits for Brazilian clinical practice as it serves as a brief and easy-to-administer tool for identifying PC needs. However, the literature indicates that there are barriers preventing this identification in a timely manner, such as denial on the part of healthcare professionals, increased workload, insufficient training to meet the needs of patients and their families, as well as institutional resistance to the adoption of integrated care practices (Gómez-Batiste et al. 2018).

To facilitate this practice, IDPALL-BR stands out as a tool designed for interprofessional practice, with the aim of fostering discussion around therapeutic planning, seeking to enhance the quality of care and, consequently, improve the quality of life of patients and their families – aspects that are intrinsically linked to the recommendations of the WHO and the National Palliative Care Program (PNCP) (Brasil 2024).

Furthermore, as this tool is not directed toward medical diagnoses, it fosters the autonomy of nursing professionals – who frequently act as the link within the healthcare team regarding care directions – enabling agility and effectiveness in the PC provided across different clinical contexts, and strengthening their role in person-centered care (Martins Pereira et al. 2021).

The methodological and contextual complexities inherent to the CCA process are among the study’s limitations, together with the dichotomous nature of the ID-PALL© instrument. The binary response structure (yes/no) may restrict the variability of respondents’ perceptions, limiting sensitivity in capturing nuances and potentially leading to inaccuracies in situations of neutrality or uncertainty. Furthermore, as it is an emerging instrument, there are no primary studies or systematic reviews addressing its use (Karagkounis et al. 2025), making it difficult to discuss specific data.

The IDPALL-BR is a promising tool regarding patient triage and stratification for the need for general and specialized PC, aligning with international recommendations for the provision of adequate care. Although its validity has been established, its wider implementation is necessary to assess its impact on clinical practice and the triage of patients with life-threatening illnesses.

## Data Availability

The data that support the findings of this study are available from the corresponding author upon reasonable request.

## Figure 1 Long description

The flowchart outlines the translation and cross-cultural adaptation process of the ID-PALL© tool across four stages. Stage 1 involves the original tool in French, identification of patients, synthesis of translations and authors′ approval for CCA of ID-PALL (F). Stage 2 includes back-translation with two versions (RT1 and RT2) and approval of Version T12. Stage 3 features the experts committee and adjustments in the pre-final version generated. Stage 4 involves pre-test, adjustments in the pre-final version consolidated and the final document in Brazilian Portuguese after original approval. The process includes identification of patients in Brazilian Portuguese.

Navigate back to Figure 1.

## Table 2 Long description

Content validity ratings from 11 experts are summarized for 15 items across four domains: semantic, idiomatic, cultural, and conceptual. For each item and domain, the table lists the average rating, error (all shown as 0.00), and the content validity coefficient (CVCC), plus an overall CVCT per item. Items 1 and 2 have perfect domain CVCC values of 1.00 and item CVCT of 1.00. Most items show strong validity, with domain CVCC commonly between about 0.93 and 1.00 and item CVCT typically around 0.94 to 0.98. Item 11 is the weakest overall, with lower domain CVCC values (notably cultural 0.86 and conceptual 0.89) and the lowest item CVCT of 0.90. The summary row indicates domain-level CVCT values of about 0.97 for semantic, 0.95 for idiomatic, 0.95 for cultural, and 0.94 for conceptual, suggesting semantic validity is highest and conceptual is slightly lower. One value in item 3 under idiomatic average appears inconsistent with the rating scale (listed as 9.82), so that entry may be a data error and should be verified.

Navigate back to Table 2.

## Table 1 Long description

Bilingual comparison of ID-PALL screening criteria, with the French original in the left column and the Portuguese T12 translation in the right column. It includes two sections: ID-PALL G with four main prompts and a set of subcriteria under item two, and ID-PALL E with eight prompts. ID-PALL G covers the “would you be surprised if the patient died within the next twelve months” question, progressive illness with general functional decline, recent marked instability, psychosocial or existential distress, need for decision-making support near end of life, possible stopping of curative or life-sustaining treatments, and requests for comfort or palliative care. The instability subcriterion lists examples such as uncontrolled symptoms, severe pressure injury, repeated acute confusion, infection, unplanned hospitalization, or falls. ID-PALL E focuses on severe persistent symptoms not adequately relieved within forty-eight hours, difficulty assessing physical or psychosocial issues, disagreement or uncertainty about treatments or resuscitation decisions, severe psychosocial or existential suffering in patients or relatives, consideration of palliative sedation, difficulty establishing advance care plans, and potential benefit from specialist palliative care. Content is presented as equivalent wording across languages rather than numerical results, so the main comparison is translation alignment, not frequency or outcomes.

Navigate back to Table 1.

## Table 3 Long description

Two-column comparison of Version T12 versus a pre-final version for three Portuguese items about psychosocial or existential distress and advance care planning. For patient distress, T12 describes severe suffering and includes examples such as anxiety or depression, isolation or feeling like a burden, loss of meaning or hope, desire to die, and a request for assisted suicide; the pre-final version shifts “severe” to “serious,” intensifies symptom wording, and removes the assisted-suicide request example. For family distress, T12 again uses “severe” and lists anxiety or depression, major exhaustion, significant family-system destabilization, and loss of meaning or hope; the pre-final version changes “severe” to “serious” and slightly streamlines the phrasing of loss of meaning and hope. For advance care planning, both versions state that establishing plans or directives with the patient and or family can be difficult, while the pre-final version expands and standardizes the terminology by naming advance care planning and advance directives explicitly and adding abbreviations. Overall, the changes are primarily editorial and definitional, aiming for consistent severity language and clearer examples rather than altering the underlying concepts.

Navigate back to Table 3.

## Table 4 Long description

Item-level content validity is summarized using average ratings, an item coefficient (CVCi), an error term, and a corrected coefficient (CVCc) for 15 items plus an overall total. Corrected CVCc values are consistently high, spanning from 0.817 (item 6) to 0.958 (item 15). Most items cluster between about 0.90 and 0.95, including item 1 at 0.95 and items 11 and 12 at 0.933. Several items share the same corrected value, such as items 4, 5, and 9 at 0.917, and items 8, 10, and 13 at 0.925. Average ratings range from 3.267 (item 6) to 3.833 (item 15), mirroring the pattern in corrected coefficients. The overall row reports an average of 3.638 and a corrected total coefficient of 0.909, indicating strong content validity across the set. Error values are extremely close to zero for each item, so differences among items are driven mainly by the average ratings and coefficients rather than the error term.

Navigate back to Table 4.

## Table 5 Long description

Two sections provide yes or no screening items to identify patients who may need general palliative care and those who may need specialist palliative care. The general section asks whether death within the next 12 months would be surprising, then whether the patient has life-limiting progressive illness plus one or more indicators: overall functional decline, marked clinical instability in the last 6 months, psychosocial or existential distress in the patient or family, or need support with end-of-life decisions. It also asks about stopping or planning to stop disease-modifying treatment or life-sustaining measures such as mechanical ventilation, dialysis, or nutrition and hydration, and whether the patient, family, or clinicians request comfort-focused care. The specialist section lists triggers including severe persistent symptoms not adequately responding within 48 hours, difficulty assessing physical or psychosocial or spiritual problems, disagreement or uncertainty about treatments or resuscitation decisions, severe psychosocial or existential distress in the patient, severe distress in family members, planned palliative sedation for an intolerable refractory symptom, difficulty establishing advance care planning or advance directives, and perceived benefit from specialist palliative care involvement. No responses are filled in, so the content functions as a structured checklist rather than reporting results or frequencies. Some criteria use time windows and severity thresholds, which require clinical judgment and local definitions when applied.

Navigate back to Table 5.
